# Draft genome sequences of nine *Bacillus* spp. strains isolated from Lebanese soil

**DOI:** 10.1128/mra.00539-26

**Published:** 2026-09-02

**Authors:** Marc Moussan, Mariam El Arab, Nancy Fayad, Mireille Kallassy Awad

**Affiliations:** 1 Laboratory of Biodiversity and Functional Genomics, UR-EGP, Université Saint-Joseph de Beyrouth 36925, Beirut, Lebanon; 2 Multi-Omics Laboratory, School of Pharmacy, Lebanese American University 114792, Byblos, Lebanon; Indiana University Bloomington, Bloomington, Indiana, USA

**Keywords:** *Bacillus thuringiensis*, genome sequencing, Illumina, bioactivity, *Bacillus mycoides*

## Abstract

The *Bacillus cereus* group is ecologically diverse but genomically close and is known for its variety of capacities, including its antimicrobial potential. Within a project aiming to find new bioactivity, nine *Bacillus* strains were sequenced: eight *Bacillus thuringiensis* and one *Bacillus mycoides*. Here, we report their genome sequences and various genomic features.

## ANNOUNCEMENT

**Fig 1:**
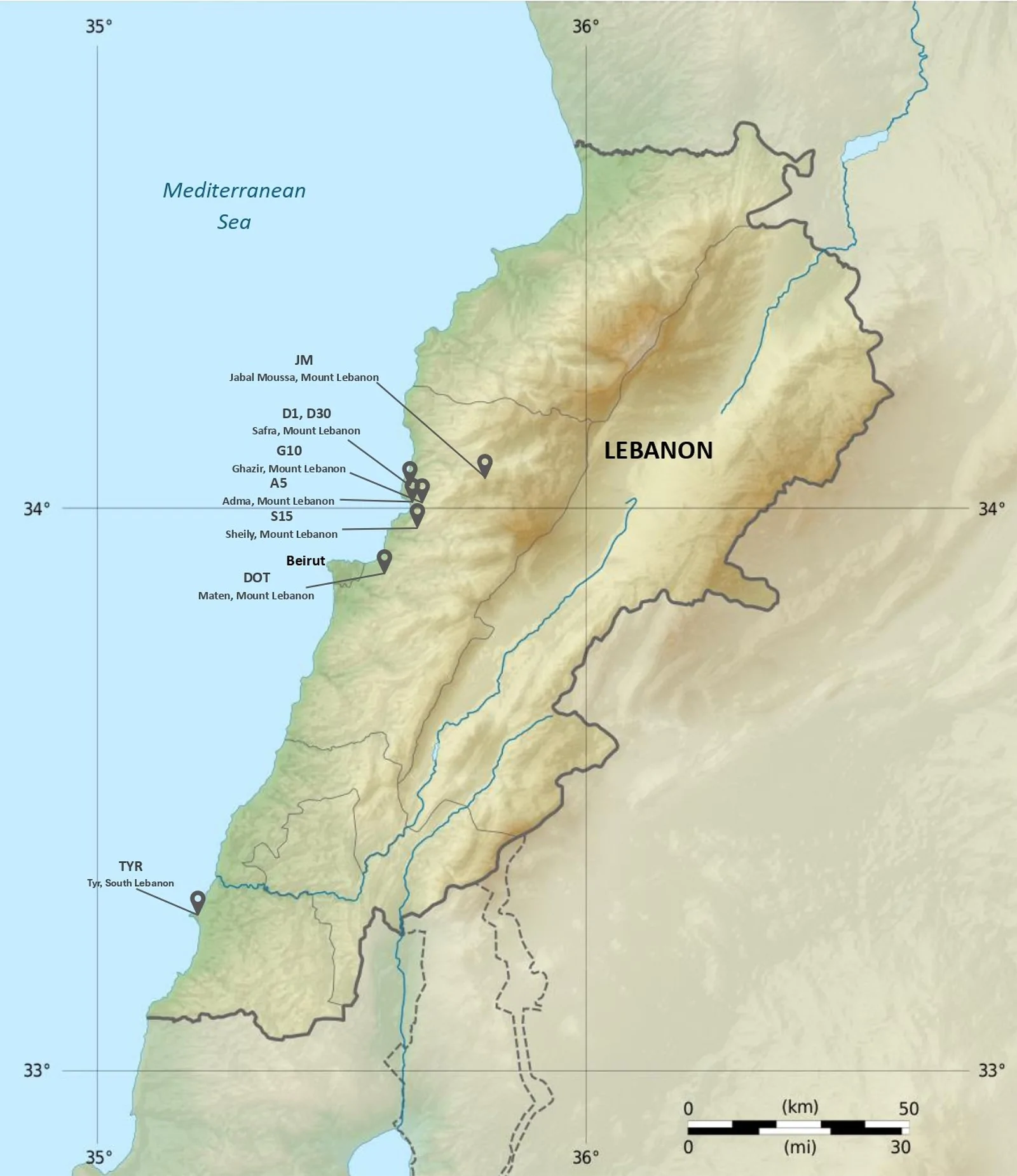
Map of Lebanon with the regions from which each strain was isolated. Reprinted from Wikimedia Commons (https://commons.wikimedia.org/wiki/File:Lebanon_location_map_Topographic.png) under a CC BY-SA 3.0 license. Map created by Eric Gaba (Sting) and NordNordWest.

*Bacillus cereus sensu lato* (*B. cereus s.l*.) is a group of gram-positive spore-forming phylogenetically close, but ecologically diverse bacterial species, with an underappreciated aspect: their antimicrobial potential (1–3). This includes, for example, the entomopathogenic *B. thuringiensis* (4, 5) and the less-studied psychrotrophic *B. mycoides* (6). Nine *Bacillus* strains were isolated from Lebanese soil per the methods described in El Khoury et al. (7). Briefly, soil samples were collected from various Lebanese regions (Fig. 1). These samples were heated in sterile water for 10 min at 80°C and diluted 10 times, after which 100 μL were plated on a sporulating medium: T3 (8). Plates were incubated at 30°C for 24 h. Matt colonies with undulated edges were replated and incubated for 48 h, then screened for the presence of parasporal crystals.

These strains were sequenced using the Illumina NovaSeq 6000 platform (Illumina, San Diego, USA) using the MicrobesNG (Birmingham, UK) standard pipeline. For DNA extraction, following overnight culture in LB medium, bacterial cell suspensions were lysed in Tris-EDTA containing 0.1 mg/mL lysozyme and RNaseA at 37°C for 25 min, then with proteinase K (0.1 mg/mL) and SDS (0.5%) at 65°C for 5 min. DNA was purified using SPRI beads, resuspended in EB buffer, quantified using the Quant-iT dsDNA HS assay (ThermoFisher Scientific), and diluted for library preparation. Libraries for sequencing were prepared using the NexteraXT Library Prep Kit (Illumina, San Diego, USA) following the manufacturer’s protocol with the following modifications: input DNA is increased twofold, and PCR elongation time is increased to 45 s. Libraries were sequenced using a 250 bp paired-end protocol. Resulting reads were trimmed using Trimmomatic v0.3 with a sliding window and quality cutoff of Q15 (9) and assembled using SPAdes v3.7 (10). Genomes were submitted to NCBI GenBank under BioProject PRJNA611745. BioSample and GenBank accessions are shown in Table 1. The number of contigs per strain ranged between 82 and 514 contigs, with an average total length of 6,321,933 bp and GC content of 34.77%. Plasmids were identified via a database similarity search using a nucleotide BLAST+ executable (v.2.16.0+ [11]) of the assembled contigs against the plasmid repertoire of the *B. cereus s.l*. group.

**TABLE 1:** Sequence features and accession numbers of the nine *Bacillus* strains

Strain	Species	Contigs^*a*^	Bioproject/sequence read project	Biosample	GenBank accession/SRA accession	Total length (bp)	# CDS^*b*^	GC content (%)	Completeness (%)^*c*^	N50/L50	Plasmid detected (yes/no)	# Reads
DOT	*Bacillus mycoides*	211 (114)	PRJNA611745/SRP684806	SAMN55937335	JBVOCQ000000000/SRR37676265	6,353,794	6,585	35.11	99.8	182789/10	Yes	1,313,457
D30	*Bacillus thuringiensis (Bt*)	514 (288)		SAMN55937336	JBVOCP000000000/SRR37676263	5,515,157	6,079	35.24	99.1	43596/42	Yes	1,102,971
G10	*Bt*	82 (40)		SAMN55937337	JBVOCO000000000/SRR37676262	6,492,699	6,504	34.62	99.8	431711/5	Yes	1,105,846
S15	*Bt*	315 (179)		SAMN55937338	JBVOCN000000000/SRR37676260	6,370,841	6,603	34.7	99.6	99663/20	Yes	1,567,397
TYR	*Bt*	364 (189)		SAMN55937339	JBVOCM000000000/SRR37676259	6,202,331	6,449	34.7	99.6	83836/22	Yes	1,216,964
A5	*Bt*	89 (47)		SAMN55937340	JBVOCL000000000/SRR37676264	6,544,216	6,616	34.63	99.8	417610/6	Yes	1,293,039
D1	*Bt*	88 (41)		SAMN55937341	JBVOCK000000000/SRR37676258	6,546,517	6,611	34.63	99.8	244445/5	Yes	1,148,147
JM	*Bt*	94 (39)		SAMN55937342	JBVOCJ000000000/SRR37676261	6,544,100	6,599	34.63	99.8	557251/5	Yes	1,553,289
Bz87	*Bt*	309 (177)		SAMN55937343	JBVOCI000000000/SRR37676257	6,327,745	6,653	34.7	99.6	99663/20	Yes	1,253,568

^*a*^ Number of contigs (number of contigs larger than 1,000 bp).

^*b*^ Total number of coding DNA sequences as per the Prokaryotic Genome Annotation Pipeline from NCBI.

^*c*^ Genome completeness as assessed by BUSCO v6.0.0 using bacillales_odb10.

All nine strains produced a proteinaceous parasporal crystal, confirmed by observation under a light microscope, as well as the presence of 4–5 crystal toxin-coding genes. Species identification was performed at several levels: by comparing the genomes to complete genomes of the *B. cereus s.l*. group retrieved from NCBI data sets, and by the NCBI and NLM taxonomy group, which relies on the average nucleotide identity score and compares the genome sequence against type strains from the NCBI Taxonomy Database (12, 13). Based on the latter classification, eight out of nine strains were classified as *B. thuringiensis*, whereas strain Dot was most closely related to *Bacillus mycoides* (Table 1).

Genome completeness was assessed using BUSCO v6.0 (14) and ranged between 99.1% and 99.8%. Genome annotation was done with NCBI’s automated annotation pipeline, Prokaryotic Genome Annotation Pipeline (15). These genome sequences are a gateway to identifying all potential bioactive molecules and lifestyle-dictating factors within these strains, guiding the way for wet lab and *in silico* downstream analysis, with tools such as AntiSmash (16) or BactiBase (17).

## Data Availability

Genome sequences for *Bacillus* strains are available in NCBI’s GenBank under the BioProject number PRJNA611745, BioSample numbers SAMN55937335 to SAMN55937343, and GenBank accession numbers JBVOCQ000000000, JBVOCP000000000, JBVOCO000000000, JBVOCN000000000, JBVOCM000000000, JBVOCL000000000, JBVOCK000000000, JBVOCJ000000000, and JBVOCI000000000. Raw reads are also available on the NCBI SRA database under the same BioProject number.
